# Effects of perinatal exposure to bisphenol A or S in EAE model of multiple sclerosis

**DOI:** 10.1007/s00441-023-03746-w

**Published:** 2023-02-08

**Authors:** Brigitta Bonaldo, Antonino Casile, Francesca Montarolo, Martina Bettarelli, Francesca Napoli, Stefano Gotti, GianCarlo Panzica, Marilena Marraudino

**Affiliations:** 1 Neuroscience Institute Cavalieri Ottolenghi (NICO), Regione Gonzole, 10-10043 Orbassano, Turin, Italy; 2 Department of Neuroscience “Rita Levi-Montalcini”, University of Turin, Via Cherasco 15, Turin, 10126 Italy; 3 School of Pharmacy, Pharmacology Unit, University of Camerino, Via Madonna delle Carceri, 9, Camerino, 62032 Italy; 4 Neurobiology Unit, Neurology, CReSM (Regional Referring Center of Multiple Sclerosis), San Luigi Gonzaga University Hospital, Orbassano, Italy; 5 Department of Molecular Biotechnology and Health Sciences, University of Turin, Turin, Italy; 6 Department of Oncology, University of Turin, San Luigi Hospital, Orbassano, Turin, Italy

**Keywords:** Endocrine-disrupting chemicals, Environmental risk factor, BPA, BPS, Experimental autoimmune encephalomyelitis

## Abstract

Epidemiological studies support the idea that multiple sclerosis (MS) is a multifactorial disease, overlapping genetic, epigenetic, and environmental factors. A better definition of environmental risks is critical to understand both etiology and the sex-related differences of MS. Exposure to endocrine-disrupting compounds (EDCs) fully represents one of these risks. EDCs are natural or synthetic exogenous substances (or mixtures) that alter the functions of the endocrine system. Among synthetic EDCs, exposure to bisphenol A (BPA) has been implicated in the etiology of MS, but to date, controversial data has emerged. Furthermore, nothing is known about bisphenol S (BPS), one of the most widely used substitutes for BPA. As exposure to bisphenols will not disappear soon, it is necessary to clarify their role also in this pathological condition defining their role in disease onset and course in both sexes. In this study, we examined, in both sexes, the effects of perinatal exposure to BPA and BPS in one of the most widely used mouse models of MS, experimental autoimmune encephalomyelitis (EAE). Exposure to bisphenols seemed to be particularly deleterious in males. In fact, both BPA- and BPS-treated males showed anticipation of the disease onset and an increased motoneuron loss in the spinal cord. Overall, BPA-treated males also displayed an exacerbation of EAE course and an increase in inflammation markers in the spinal cord. Analyzing the consequences of bisphenol exposure on EAE will help to better understand the role of both xenoestrogens and endogenous estrogens on the sexually dimorphic characteristics of MS.

## Introduction

Multiple sclerosis (MS) is a chronic autoimmune disease of the central nervous system (CNS), characterized by perivascular infiltration of inflammatory cells, demyelination, axonal loss, and gliosis (McGinley et al. 2021; Thompson et al. 2018). MS has a different prevalence in the sexes and the female-to-male *ratio* varies between 1.5:1 and 2.5:1 or more (i.e., 2.3–3.5:1), as reported in several studies (Harbo et al. 2013; Ortona et al. 2016; Voskuhl 2020). This data, indicating an increase in MS among women but not men, have brought about in-depth studies of sex differences in the nervous system or immune system, possibly due to genetic differences, gonadal hormones, environmental factors, and/or lifestyle (Alfredsson and Olsson 2019; Ascherio and Munger 2016; Hedström et al. 2021; Olsson et al. 2017).

Overall, women exhibit more robust responses of the immune system than men, and this is thought to influence the different susceptibility to develop autoimmune diseases (Di Florio et al. 2020; Rubtsova et al. 2015). This quick increase could reveal unrecognized environmental or nutritional changes (Alfredsson and Olsson 2019; Ascherio and Munger 2016). Sex’s effect on the clinical features of MS is unclear. Still, there are indications that women usually have earlier onset of disease, a lower prevalence of primary progressive disease course, and generally show less progression to disability than men (Bergamaschi 2007; Harbo et al. 2013; Olsson et al. 2017; Ramien et al. 2016; Voskuhl 2020).

Among the main factors affecting these sex differences, gonadal hormones and different responses to environmental factors appear to be particularly significant (Rubtsova et al. 2015). The role of sex hormones in MS appears to be limited to women; however, the situation is much more tangled (Ortona et al. 2016; Ramien et al. 2016; Shepherd et al. 2020). Because of the presence of hormone receptors on immune cells, sex hormones can affect the activities of the immune system and possibly influence different aspects of autoimmune diseases (i.e., risk, activity, and progression) (Moulton 2018). In general, estrogen and prolactin act as enhancers of humoral immunity, whereas testosterone and progesterone act as immunosuppressants (Pierdominici et al. 2010; Shepherd et al. 2020). The effects mediated by the estrogens depend on the dose: lower levels stimulate specific immune activities, while higher levels, for example, those observed in pregnancy, inhibit them (Merz et al. 2022; Whitacre et al. 1999). Hence, the different effects mediated by the sex hormones depend not only on the concentration but also on the cell type and receptor subtype expressed in a given cell type (Ortona et al. 2016).

In view of the wide range of effects that sex hormones play within the CNS (Spence and Voskuhl 2012), it is possible to hypothesize these hormones have a role in MS, acting not only on immune system cell populations but also on the CNS ones (Spence and Voskuhl 2012; Ysrraelit and Correale 2019): some endogenous and exogenous estrogens are useful in MS patients both during pregnancy (Gilli et al. 2010; Merz et al. 2022) and when using oral contraceptives (Chen et al. 2020; Sena et al. 2012). However, their action appears to be effective only in the early stages of MS (Spence and Voskuhl 2012). Estrogen can also act on astrocytes which modulate neuronal death and inflammation through several pathways. The action of estrogens is mediated through the estrogen receptor α (ERα), which reduces inflammation, demyelination, and axonal loss (Spence et al. 2011; Tiwari-Woodruff et al. 2007; Yilmaz et al. 2019), while the estrogen receptor β (ERβ) has a more controversial role. It is not involved in endogenous estrogen protection but can respond to exogenous ligands, protecting against demyelination and axonal loss and stimulating endogenous myelination (Crawford et al. 2010; Spence and Voskuhl 2012). In women, studies are underway for estriol treatment with anti-inflammatory, neuroprotective, and immunomodulatory effects (Voskuhl et al. 2016). The role of ERβ in MS is less known, but it is an attractive therapeutic candidate in association with some anti-inflammatory drugs. Indeed, estriol binds to ERα and ERβ weaker than estradiol, but it shows a higher affinity to ERβ compared to ERα, the main cause of estrogenic effects on breast cancer and cardiovascular diseases (Voskuhl et al. 2016). Interestingly, a more recent study performed in the experimental autoimmune encephalomyelitis (EAE) model of MS demonstrates that, in this model, along with inflammation and demyelination in the spinal cord, it presents inflammation of the hypothalamic tissue in both females and males. This inflammation results in the downregulation of different genes in males and females, leading to sex-specific changes downstream in the hypothalamic-pituitary axis (HPA) (Milosevic et al. 2020), supporting the idea that EAE partially also models sex-specific characteristics of the disease (Ryan and Mills 2021).

Along with endogenous estrogens, the organism can be exposed to natural (phytoestrogens and mycoestrogens) or synthetic (xenoestrogens) compounds with estrogenic activity, i.e., endocrine-disrupting compounds (EDCs). There are thousands of chemicals, such as plastics, dust, pesticides, herbicides, and medical and/or dietary components, that have been classified as EDCs (Gore et al. 2015; Ho et al. 2022). Exposure to EDCs is more perilous if it occurs during peculiar “critical periods” of life (e.g., intrauterine, perinatal, juvenile, or puberty periods) when organisms are more sensitive to hormonal action. Nevertheless, exposure to EDCs in adulthood also can alter physiology (Frye et al. 2012; Kahn et al. 2020).

Synergic/additive effects might be displayed by environmental and endogenous estrogens, potentially also affecting the immune response. Moreover, there is numerous in vitro and in vivo evidence that these compounds may exert immunotoxic effects (Chighizola and Meroni 2012; Ortona et al. 2016). Many studies also highlighted the issues due to EDC exposure on steroid hormone receptors in the developing CNS, showing substantial effects on mRNA levels, protein expression, and neuroanatomical and functional consequences of altered receptor action (Gore et al. 2015).

There is evident crosstalk between the CNS and the immune system. In fact, on the one hand, lymphoid organs present receptors for neuropeptides, neurotransmitters, and hormones, and on the other one, immune system activation causes changes in hypothalamic, autonomic, and endocrine functions (Bahadar et al. 2015; Del Rey and Besedovsky 2017). Moreover, immune functions are also modulated by the interplay between the autonomic and the neuroendocrine systems via the pituitary-adrenal axis, which represents a crucial link between CNS-immune interaction and autoimmune diseases (Bahadar et al. 2015). Therefore, exposure to EDCs could increase the risks or intensify the aggressiveness of autoimmune diseases affecting the CNS, above all MS (Ascherio et al. 2012).

Bisphenol A (BPA) is a compound mainly used for the production of clear and tough plastics, utilized for the manufacturing of many common consumer goods (Abraham and Chakraborty 2020). It represents one of the most known and studied EDCs (Abraham and Chakraborty 2020). Exposure to BPA has been described to alter the function of some systems, including the immune system (Kimber 2017; McDonough et al. 2021; Rochester 2013). In fact, in mice, BPA exposure resulted in augmented production of cytokine and antibodies and decreased numbers of regulatory T cells, even if many of the reports focused on adult, as opposed to gestational, exposure to this compound (Kimber 2017; Rochester 2013). The risk to public health due to BPA exposure was recognized by EFSA in 2015 (EFSA, 2015): the tolerable daily intake (TDI) for BPA was reduced from 50 to 4 µg/kg body weight/day, but the BPA substitutes, such as bisphenol S (BPS), have no specific limitations, even if they seem to have comparable, or even more alarming, endocrine disrupting properties as the BPA (den Braver-Sewradj et al. 2020; Eladak et al. 2015; Gramec Skledar and Peterlin Masic 2016; McDonough et al. 2021; Rochester and Bolden 2015).

Data on the effects of BPA exposure on different MS animal models appear to be particularly controversial, either excluding (Krementsov et al. 2013) or supporting (Brinkmeyer-Langford et al. 2014; Rogers et al. 2017) its potential effects on peculiar aspects of the disease.

Considering the increasing exposure to EDCs, and in particular to BPs, and that the environmental components which have been implicated in the etiology of MS, it is important to properly examine their role in the onset and course of the disease. Thus, taking the advantage of the EAE mouse model of MS, this study aimed to better understand the consequences of perinatal exposure to BPA and to evaluate and compare the one of BPS, in mice of both sexes. We assessed daily the severity of the disease both by carrying out a clinical evaluation and testing the motor symptoms, evaluating the performance with the rotarod. Finally, we evaluated the degree of inflammation and the motoneuron loss thanks to histological investigations.

## Materials and methods

### Animals

Adult C57BL/6 J mice from our colony at the Neuroscience Institute Cavalieri Ottolenghi (originally purchased from Envigo, S. Pietro al Natisone, Udine, Italy) were housed in standard conditions in 45 × 25 × 15 cm polypropylene mouse cages at 22 ± 2 °C, under 12:12 light–dark cycle (lights on at 08:00 AM). Food (standard mouse chow 4RF21, Mucedola srl, Settimo Milanese, Italy) and water were provided ad libitum. One male and two female mice (3-month-old) were housed together to achieve a successful mating, assessed by the evaluation of the presence of the vaginal plug (assumed as gestational day 0, GD0) (Hasegawa et al. 2017).

The experimental design conforms to the ARRIVE guidelines originally published by Kilkenny et al. in 2010 (2010).

### Treatments

BPA (Sigma-Aldrich, 239,658, CAS 80–05-7) or BPS (Sigma-Aldrich, 103,039, CAS 80–09-1) were prepared for oral administration by dissolving them in corn oil (Sigma-Aldrich, C8267). Twelve pregnant dams were assigned randomly to three experimental groups: oil-treated dams (receiving only vehicle, corn oil; *n* = 4), BPA-treated dams (receiving 4 µg/kg BW/day of BPA, corresponding to the European TDI; *n* = 4), and BPS-treated dams (receiving 4 µg/kg BW/day of BPS; *n* = 4). The dose was calculated daily according to the dams’ body weight, recorded with an electronic precision balance (Mod. Kern-440-47N, resolution 0.1 g).

We decided to test the same dose for both BPA and BPS to allow a precise comparison of the effects of the two bisphenols. Moreover, at present, although BPS is one of the most used BPA substitutes and it has already been detected in environmental and human samples (Catenza et al. 2021), at present, no user guidelines are available. Dams were treated starting at GD0, throughout pregnancy and lactation, until weaning of the offspring at postnatal day 28 (PND28). To resemble human exposure conditions, the daily treatment or the vehicle was given orally to the dams by means of a pipette to minimize the dams’ stress (Bo et al. 2016; Palanza et al. 2002). This type of administration allowed us to perform a perinatal treatment (covering both prenatal and postnatal critical windows of development) (Neier et al. 2019) on the offspring. In fact, it is known that both BPA and BPS can pass first through the placenta and then into the milk during lactation (Cimmino et al. 2020; Mao et al. 2020).

Litters were reduced to 8 pups at birth to obtaining an equal number of pups of both sexes, sexed via the measurement of the anogenital distance (AGD) (Manno 3rd, 2008). The pups were weaned at PND28 and housed in monosexual groups of 4 mice. They were monitored weekly until adulthood (PND56), when the experimental procedures were performed.

### EAE induction and clinical evaluation

Chronic EAE has been induced in 8 weeks-old mice of both sexes (*n* = 9/group) (Constantinescu et al. 2011). Briefly, mice have been immunized by subcutaneous immunization under the rostral part of the flanks and at the base of the tail with 300 µl of 200 µg/mouse of myelin oligodendrocyte glycoprotein (MOG_35–55_; Espikem, Florence, Italy) in incomplete Freund’s adjuvant containing 8 mg/ml of *Mycobacterium tuberculosis* (strain H37Ra; Difco Laboratories Inc., St Henry, Detroit, Michigan, USA) and two intravenous injections of 500 ng of Pertussis toxin (Duotech, Milan, Italy) the day of immunization and 48 h after (i.e., 2 days post-immunization, dpi) (Montarolo et al. 2014, 2015).

Body weight (BW) and clinical score (CS; 0, healthy; 1, limp tail; 2, ataxia and/or paresis of hind limbs; 3, paralysis of hind limbs and/or paresis of forelimbs; 4, tetra paralysis; 5, dying or death) have been recorded daily by a blind investigator. This analysis allows to evaluate the clinical differences in the onset and progression of the disease (Constantinescu et al. 2011; Montarolo et al. 2014, 2015).

Furthermore, since the rotarod test could be used as a more quantitative and precise clinical assessment of the disease course than the clinical score alone (van den Berg et al. 2016), mice underwent a rotarod performance test daily (Mouse RotaRod, Ugo Basile 47600, Milan, Italy), starting from 6 dpi until the time of the sacrifice (28 dpi). The 1–5 dpi period has been used to train the animals in the use of the device and to obtain reference values (baseline). The test consisted of a single 300 s session during which the rod speed was increased linearly from 4 to 40 rpm (van den Berg et al. 2016). When the mouse was not capable of maintaining its balance and fell off the device, it fell and triggered a sensor, and the time (s) was recorded (latency).

Within the four weeks (0–28 dpi) of EAE follow-up, we also monitored the food intake (FI, g/animal/day; once a week), and, in females, we checked the estrous cycle for at least 2 cycles, evaluating the vaginal cytology smears (McLean et al. 2012). In particular, we focused on the evaluation of the estrous cycle between the first and the second week post-immunization (considering, in particular, the acute phase of the EAE) (Constantinescu et al. 2011) because it has been demonstrated that the immunization procedure causes the greatest alterations of the estrous cycle within this period (Rahn et al. 2014).

### Fixation and tissue sampling

At 28 dpi, mice were sacrificed by deep irreversible anesthesia (intraperitoneal injection of Zoletil 80 mg/kg/ Rompum 10 mg/kg) and transcardially perfused with 4% paraformaldehyde (PFA) solution. Spinal cords were removed and stored in a 4% PFA solution for 24 h, followed by several washes in 0.01 M saline phosphate buffer (PBS). Finally, they were embedded in paraffin. Paraffin-embedded spinal cords were cut in the transversal plane at 10 µm thickness with a microtome and collected on gelatin-coated slides. The plane of sectioning was oriented to match the drawings corresponding to the transversal sections of the mouse spinal cord atlas (Watson et al. 2009).

### Histological evaluations

Ten-µm-thick paraffin-embedded sections on gelatin-coated slides, representative of the entire spinal cord, were stained with Hematoxylin–Eosin (Montarolo et al. 2014, 2015) or Cresyl Violet (Nissl Staining) (Morales et al. 2006), to detect the presence of the perivascular inflammatory infiltrates (PvIIs; *n* = 9 animals/group) and the motoneurons (MNs; *n* = 5 animals/group), respectively. The presence of PvIIs and MNs loss is assessed as signs of the disease (Bolton and Smith 2015; Constantinescu et al. 2011; Frezel et al. 2016; Gushchina et al. 2018).

Briefly, the staining was performed as follows: after deparaffinization, sections were stained with the Hematoxylin and Eosin procedure by using Sigma-Aldrich (St. Louis, Missouri, USA) reagents, or they were Nissl-stained with 0.1% Cresyl Violet (Sigma-Aldrich, St. Louis, Missouri, USA). Dehydrated sections were covered with New-Entellan mounting medium (Merck, Milano, Italy).

### Quantitative analysis

Neuropathological findings were quantified in 10 complete cross-sections of the spinal cord per mouse representative of whole spinal cord levels. The sections were acquired and analyzed with the Neurolucida software connected to an E-800 Nikon microscope with a 20 × objective (Glaser and Glaser 1990). The number of PvIIs or MNs was calculated and expressed as the number of PvIIs or MNs per mm^2^.

The representative images in Figs. 3 and 4 were acquired with a NIKON DS-U1 digital camera (Software of acquisition: NIS-Element AR 2.10) connected to a NIKON Eclipse 90i microscope (Nikon Italia S.p.S., Firenze, Italy). Images were digitized by using a 10 × or 40 × objective for the acquisition.

### Statistical analysis

BW, FI, CS, and latency at the rotarod performance test were analyzed by three-way (sex, treatment, and time as independent variables) analysis of variance (ANOVA). All other quantitative data were analyzed by two-way (sex and treatment as independent variables) ANOVA with SPSS 27 statistic software (SPSS Inc., Chicago, USA). If the ANOVA was significant, the post hoc analysis was performed using Tuckey’s HSD test. Comparison between the estrous cycle evaluations was performed using the Student’s *t* test. Differences were considered statistically significant for values of *p* ≤ 0.05. Data are shown as mean ± SEM (mean standard error).

## Results

### Effects of BPs on body weight, food intake, and estrous cycle of EAE-affected mice

**Fig. 1:**
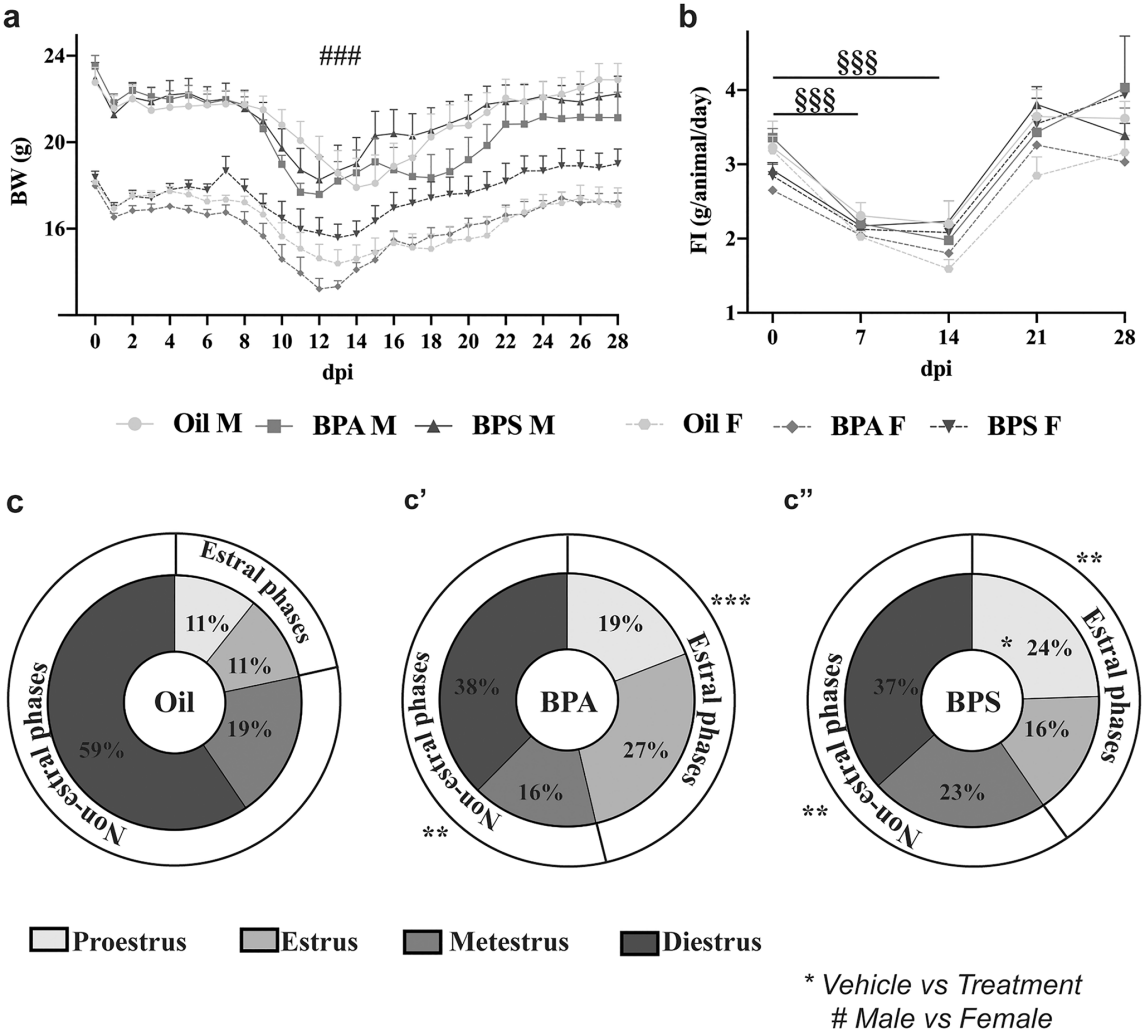
Effects of BPA and BPS exposure on body weight, food intake, and estrous cycle of EAE-affected mice. Daily body weight (**a**) and weekly food intake (**b**) evaluation from the day of immunization (0 dpi) until the sacrifice (28 dpi) of the animals. Mean percentage of time spent in the different phases of the estrous cycle, assessed by vaginal cytology smears, in the (**c**) oil-, (**c**’) BPA-, or (**c**”) BPS-treated EAE-affected females. Data are expressed as mean ± SEM. Statistical analysis revealed a significant effect for *p* ≤ 0.05 (* = vehicle vs. treatment; # = male vs. female; § = comparison between different timepoints). BW, body weight; FI, food intake; dpi, day post-immunization

The analysis of BW showed no effects of the treatments (*F*_*(2,48)*_ = *0.472*, *p* = *0.627*; Fig. 1a). However, the sex differences in BW (*F*_*(2,48)*_ = *1.498*, *p* = *0.011*; Fig. 1a) were always maintained (*p* < *0.001*; Fig. 1a) despite the treatments and the disease progression. Finally, all the experimental groups displayed a similar BW trend, showing a decrease (Fig. 1a) in the acute phase (within the second week post-immunization) of the disease due to the increased EAE severity.

The analysis of FI did not show any differences between the groups (*F*_*(2,8)*_ = *0.950*, *p* = *0.485*; Fig. 1b) but highlighted a significative decrease among all groups within the first and second post-immunization week (*p* < *0.001*), due to the increased EAE severity which caused difficulties in reaching the food placed in an upper container in the cage. To avoid further stress on the animals, the food was then placed on the cage ground, so the FI returned to starter levels, and the BW partially recovered (Fig. 1b).

The analysis of the estrous cycle in EAE-affected females revealed that both BPA and BPS treatments caused an alteration in the time spent in the different phases of the estrous cycle (Fig. 1c, c’, c”). Both BPS-treated females spent more time in estral phases (proestrus and estrus; oil vs. BPA*, p* < *0.001, *Fig. 1c’; oil vs. BPS, *p* = *0.004, *Fig. 1c”) and less in non-estral ones (metestrus and diestrus; oil vs. BPA*, p* = *0.008, *Fig. 1c’; oil vs. BPS, *p* = *0.004, *Fig. 1c”) compared to oil-treated ones (Fig. 1c). In particular, BPS-treated females spent more time in proestrus compared to oil-treated females (*p* = *0.031*; Fig. 1c”).

### Effects of BPs on EAE onset and course

**Fig. 2:**
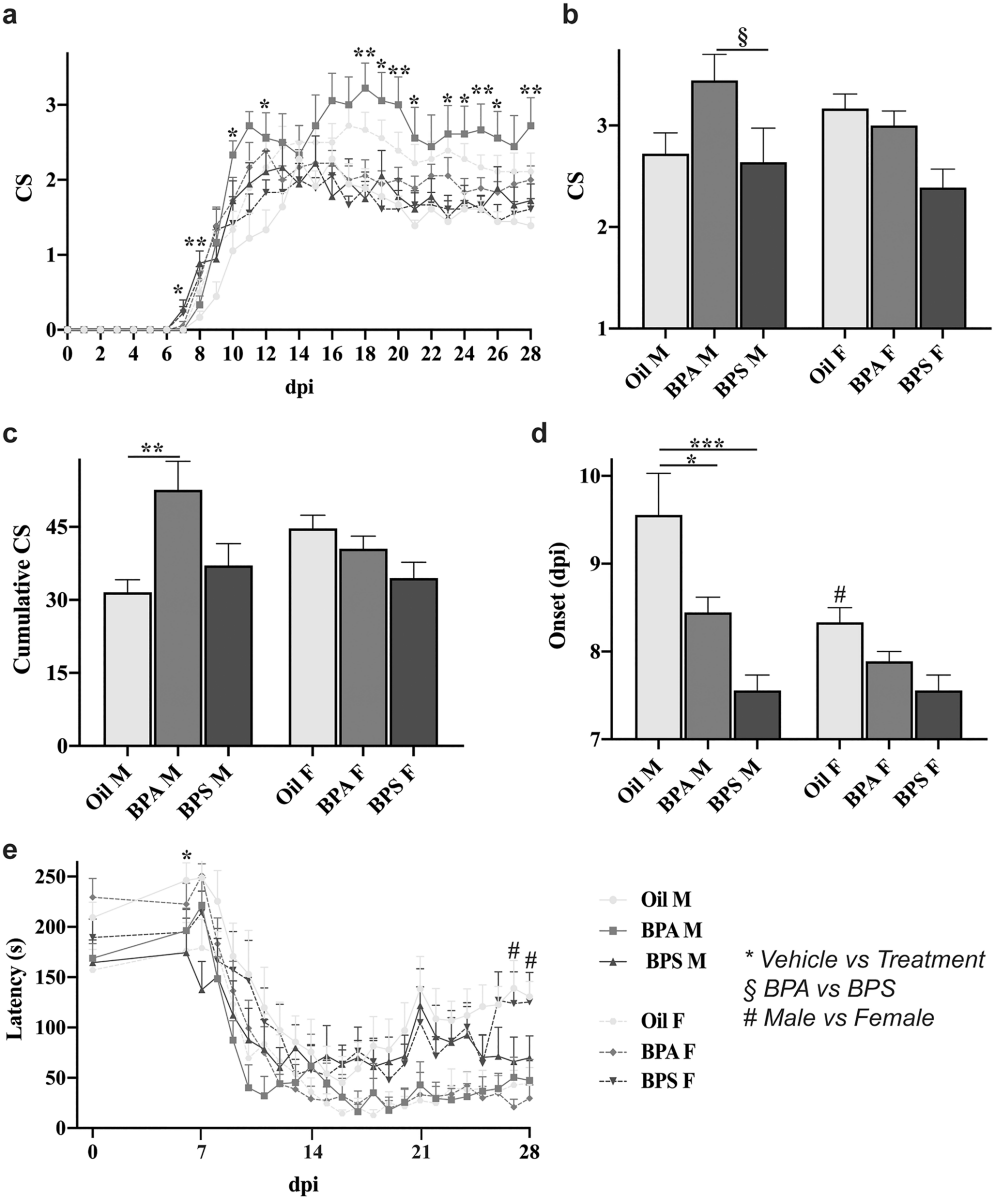
Effects of BPA and BPS exposure on EAE clinical evaluations. **a** Daily clinical score evaluation from the day of immunization (0 dpi) until the sacrifice (28 dpi) of the animals. Mean **b** maximum clinical score and **c** cumulative clinical score reached by the oil- (*left*), BPA- (*center*), or BPS- (*righ*t) treated EAE-affected male (*left side of the graph*) and female (*right side of the graph*) mice. **d** Mean dpi of disease onset in the oil- (*left*), BPA- (*center*), or BPS- (*righ*t) treated EAE-affected male (*left side of the graph*) and female (*right side of the graph*) mice. **e** Daily evaluation of rotarod performance (expressed as latency of fall) from 6 dpi (the 0 represents the baseline values obtained within the first 5 days of the test) until the sacrifice (28 dpi) of the animals. Data are expressed as mean ± SEM. Statistical analysis revealed a significant effect for *p* ≤ 0.05 (* = vehicle vs. treatment; § = BPA vs. BPS; # = male vs. female). CS, clinical score; dpi, day post-immunization

The clinical evaluation of the EAE course was assessed daily, assigning both the CS and evaluating the rotarod performance (as latency of fall) in all experimental groups (Fig. 2).

First, the analysis of the daily CS showed some significant differences in the disease course (*F*_*(2,48)*_ = *6.481*, *p* = *0.003*; Fig. 2a). That is due to an increased CS among the BPA-treated males, which also displayed higher maximum reached CS (Fig. 2b) and a significantly higher cumulative CS (*p* = *0.004*, Fig. 2c) compared to oil-treated males. Interestingly, BPS-treated males displayed a significant increase in CS, compared to oil-treated ones, only at 7 dpi (*p* = *0.044*) and 8 dpi (*p* = *0.004*). Furthermore, both BPA- (*p* = *0.027*) and BPS- (*p* < *0.001*) treated males showed anticipation in disease onset (Fig. 2d) compared to the oil-treated ones, which disrupted the sexual dimorphism existing among the oil-treated mice where the females displayed an anticipated onset compared to males (*p* = *0.011, *Fig. 2d).

The analysis of the daily rotarod performance showed some significant differences among groups (*F*_*(2,48)*_ = *4.069*, *p* = *0.023*; Fig. 2e) due to the fact that BPS-treated males displayed lower latency at 7 dpi (*p* = *0.020*) compared to oil-treated ones. Among oil-treated groups, females showed lower latency at 27 dpi (*p* = *0.020*) and 28 dpi (*p* = *0.043*) compared to males.

### Effects of BPs on histological parameters in the spinal cord

**Fig. 3:**
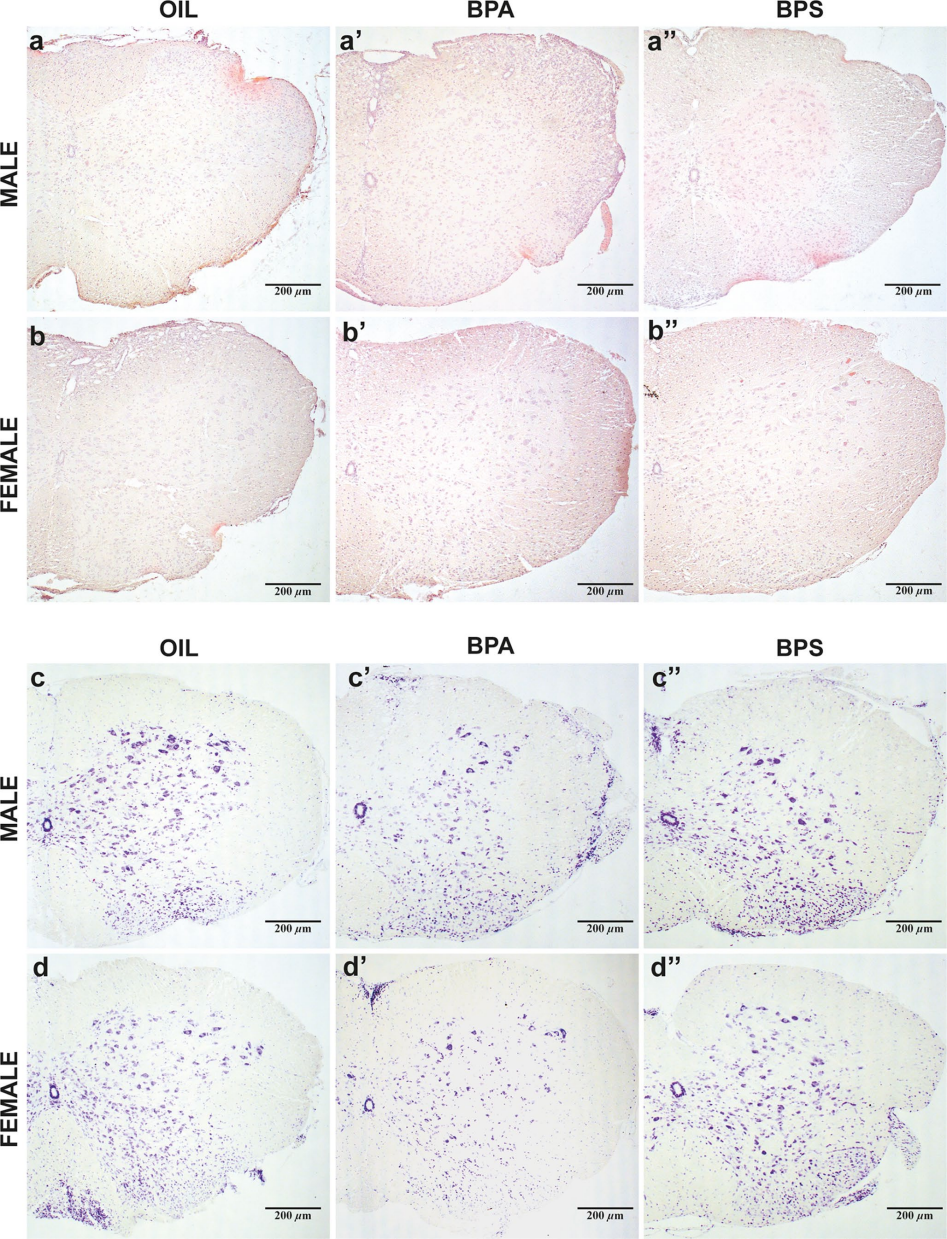
Histological staining of EAE-affect mice’s spinal cord. Representative images of transversal spinal cord sections stained with Hematoxylin–Eosin (*upper panel*) to assess the presence of PVIIs, of oil- (*left*), BPA- (*center*), or BPS- (*right*) treated male (**a, a’, a”**) or female (**b, b’, b”**) mice. Representative images of transversal spinal cord sections stained with Cresyl Violet (*lower panel*) to assess the number of MNs, of oil- (*left*), BPA- (*center*), or BPS- (*right*) treated male (**c, c’, c”**) or female (**d, d’, d”**) mice. Scale bar = 200 µm (10 × magnification). PvIIs, perivascular inflammatory infiltrates; MNs, motoneurons

**Fig. 4:**
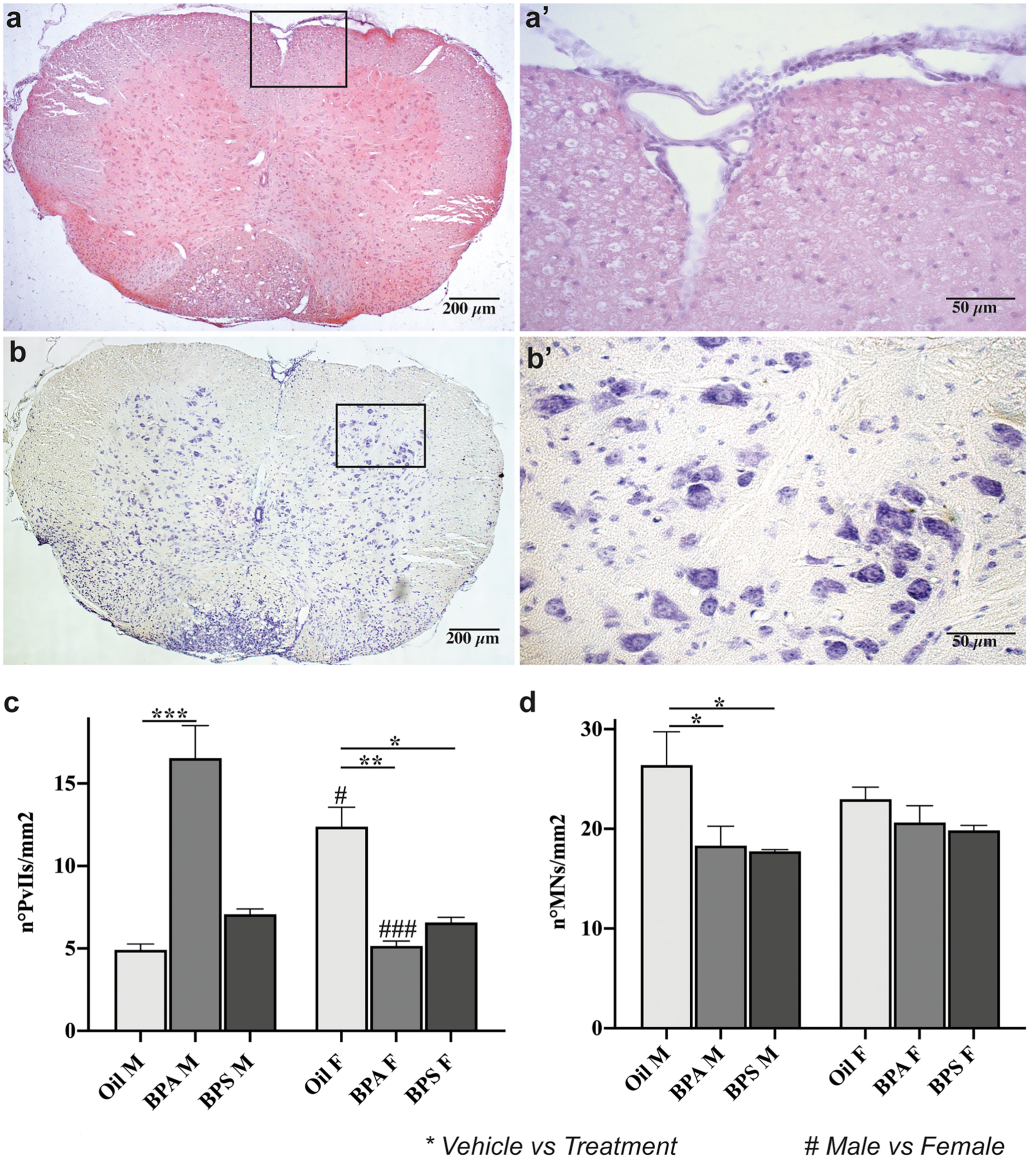
Analysis of perivascular inflammatory infiltrates and motoneuron loss in spinal cord sections of oil-, BPA- or BPS-treated EAE-affected mice. Representative images of (**a, a’**) Hematoxylin–Eosin and (**b, b’**) Nissl staining in a transversal section of spinal cord from an oil-treated EAE-affected male mice. Analysis of the (**c**) presence of PvIIs and (**d**) motoneuron loss in the spinal cords of oil- (*left*), BPA- (*center*), or BPS- (*right*) treated EAE-affected male (*left side of the graph*) and female (*right side of the graph*) mice. Data are expressed as mean ± SEM. Statistical analysis revealed a significant effect for *p* ≤ 0.05 (* = vehicle vs. treatment; # = male vs. female). Scale bar = 200 µm (10 × magnification) or 50 µm (40 × magnification). PvIIs, perivascular inflammatory infiltrates; MNs, motoneurons

The presence of PvIIs, observed in Hematoxylin–Eosin-stained sections of the spinal cord (*representative sections in *Fig. 3a–b”), and the MNs loss, measured in Cresyl Violet stained sections (*representative sections in *Fig. 3c–d”), are assessed as signs of disease severity (Fig. 4).

The quantification of PvIIs (Fig. 4a, a’) highlighted some significant differences among the groups (*F*_*(5,54)*_ = *12.656*, *p* < *0.001*; Fig. 4c*)*. First, oil-treated females displayed significantly higher values compared to oil-treated males (*p* = *0.013*, Fig. 4c). This sexual dimorphism was disrupted in the treated groups. In fact, BPA-treated males showed a significant increase compared to oil-treated males (*p* < *0.001*, Fig. 4c), while BPA-treated females showed a significant decrease compared to oil-treated females (*p* = *0.006*, Fig. 4c), causing an opposite and extreme sexual difference (*p* < *0.001*, Fig. 4c). On the other hand, BPS-treated males showed no difference compared to oil-treated ones (*p* = *0.999*, Fig. 4c), while BPS-treated females showed a significant decrease compared to oil-treated females (*p* = *0.033*, Fig. 4c), thus causing the disappearance of the sexual dimorphism in BPS-treated animals.

The analysis of MNs loss (Fig. 4b, b’) showed some significant differences among the groups (*F*_*(5,24)*_ = *3.189*, *p* = *0.024*; Fig. 4d*)*. In particular, both BPA- (*p* = *0.044*) and BPS- (*p* = *0.027*) treated males displayed a decreased number of MNs compared to the oil-treated ones (Fig. 4d), while we found no differences among the females (Fig. 4d).

## Discussion

MS is a multifactorial disease that overlaps with genetic, epigenetic, and environmental factors (Ascherio et al. 2012; Ascherio and Munger 2016; Waubant et al. 2019). Thus, defining the environmental risks is a crucial turning point to better understand the great variability of the diseases in terms of etiology, progression, and sexual prevalence (Ascherio et al. 2012; Ascherio and Munger 2016; Hedström et al. 2021).

In our study, we highlighted, in the EAE model of MS, how exposure to either BPA or BPS during a critical period of development affected the disease onset and course differentially in the two sexes. BPs treatment seemed to be particularly serious in males. In fact, BPA-treated males displayed the greatest alterations, showing a more aggressive disease in terms of anticipation of disease onset, clinical score, inflammation, and motoneuron loss in the spinal cord. Furthermore, also BPS-treated males displayed anticipation of the disease onset and a higher motoneuron loss in the spinal cord compared to oil-treated males. Among females, we did not notice any significant differences in the evaluated disease-related parameters, except for fewer PvIIs in the spinal cord, which did not come along with a recovered number of motoneurons.

Sex’s effect on the clinical features of MS is unclear. However, there are indications that women usually have an earlier onset of disease, a slightly lower prevalence of primary progressive disease course, and a minor progression to disability than men (Bergamaschi 2007; Harbo et al. 2004; Ramien et al. 2016; Voskuhl 2020). So, even if MS is more prevalent in women compared to men, men generally develop a more aggressive and progressive form of the disease (Harbo et al. 2013; Ortona et al. 2016). As environmental exposures play a role in determining those differences (Alfredsson and Olsson 2019; Ascherio and Munger 2016), our results support the idea that exposure to BPs could lead to an exacerbation of the diseases in males.

Data on the effects of BPA exposure on different MS animal models appear to be controversial, while we have no information about the BPS. A 2013 study in EAE-affected female mice did not support the hypothesis that gestational BPA exposure contributes to the increasing female MS risk (Krementsov et al. 2013). On the contrary, another 2013 study investigated, both in male and female mice, the effects of perinatal BPA exposure on Theiler’s-virus-induced demyelination (TVID), another murine model of MS, showing that perinatal BPA exposure is linked to a decreased level of viral antibodies, an anticipation of the onset of TVID symptoms, an increased inflammation in both the spinal cord and digestive tract, and an intensified changes in immune-related gene expression caused by viral infection (Brinkmeyer-Langford et al. 2014). The controversial results could be linked to the fact that MS is modeled using different animal models, which reflect only partially the characteristic of the disease (Lassmann and Bradl 2017; Procaccini et al. 2015) and to the different periods and ways of administration and dose selected for BPA treatment (Panzica and Melcangi 2016).

However, our results are in line with the work of Rogers et al. (2017), which demonstrated that gestational exposure to BPA lowered the threshold for EAE onset, especially in male mice. It is interesting to notice that we observed the deleterious effects of the exposure at a lower dose (4 µg/kg BW/day vs. 1 or 3 mg/kg BW/day). Moreover, we also highlighted the pathological signs of the disease at spinal cord levels. Finally, for the first time, we described the effect of BPS exposure in a murine model of MS. A more recent paper shows that subchronic exposure to BPA in mice led to the deregulation of inflammatory cytokines and oxidative stress, possibly linked to neurotoxicity, axonal damage, and myelin degeneration (Khan et al. 2019). This mechanism could underlie the motoneuron loss in an immune system-independent way, which seemed to be the case, especially in BPS-treated males, displaying a significant decrease in the number of motoneurons without any increase in PvIIs compared to oil-treated ones. Increasing in vitro evidence describes the neurotoxic potential of BPS (Meng et al. 2021; Pang et al. 2019), while the inflammatory potential of BPS appears to be less compared to the one of BPA (Kobayashi et al. 2010; Profita et al. 2021).

In females, we did not observe any statistically relevant effects of both BPs on disease onset and course, except for a reduction in PvIIs in the spinal cord. This could be due to the effects of BPs on the estrous cycle. In fact, both BPA and BPS led to an increase in time spent in estral phases in treated females compared to oil-treated ones. This phase is characterized by increasing levels of estrogens which are known to exert an anti-inflammatory, neuroprotective effect (Spence and Voskuhl 2012). In particular, BPS-treated females spend more time in proestrus, which has been described as protective at least against neurological symptoms in the EAE models (Rahn et al. 2014).

It is important to underline the fact that the observed alterations are present in adult animals following perinatal exposure. Perinatal BPA and BPS could cause an impairment either in immune system cell populations or in motoneurons which is maintained in adulthood or may result in an altered response to stimuli. Moreover, both BPs presumably would accumulate within some compartments of the organism and face a slow release (Charisiadis et al. 2018; Venisse et al. 2019). Finally, different effects observed in males and females could be due also to the fact that males appeared to be particularly vulnerable to developmental exposure to BPs (Kobayashi et al. 2010).

Studying the effects of exogenous compounds with estrogenic activity can contribute to a better understanding of the role of endogenous hormones and identify the mechanisms underlying sex differences in MS (Harbo et al. 2013; Ortona et al. 2016; Ramien et al. 2016; Voskuhl 2020). Furthermore, a better definition of environmental risks is necessary. Investigating the effects of BPs exposure can support better determining their deleterious properties, which may be particularly relevant in pathological conditions. Additionally, defining BPs as a real risk of developing or worsening MS can help devise new strategies to reduce exposure for sensitive people or patients (e.g., avoiding specific environments, do not use plastic food/water containers, etc.).

## Data Availability

All data generated or analyzed during this study are included in this article. Further enquiries can be directed to the corresponding author.
